# Anti-Human VEGF Repebody Effectively Suppresses Choroidal Neovascularization and Vascular Leakage

**DOI:** 10.1371/journal.pone.0152522

**Published:** 2016-03-25

**Authors:** Da-Eun Hwang, Jeong-Hyun Ryou, Jong Rok Oh, Jung Woo Han, Tae Kwann Park, Hak-Sung Kim

**Affiliations:** 1 Department of Biological Sciences, Korea Advanced Institute of Science and Technology (KAIST), 291 Daehak-ro, Yuseong-gu, Daejeon, 305–701, Korea; 2 Graduate School of Medical Science & Engineering, Korea Advanced Institute of Science and Technology (KAIST), 291 Daehak-ro, Yuseong-gu, Daejeon, 305–701, Korea; 3 Department of Ophthalmology, Soonchunhyang University, College of Medicine, Bucheon Hospital, Bucheon, Korea; University of Illinois at Chicago, UNITED STATES

## Abstract

Age-related macular degeneration (AMD) is the leading cause of vision loss and blindness among people over the age of 60. Vascular endothelial growth factor (VEGF) plays a major role in pathological angiogenesis in AMD. Herein, we present the development of an anti- human VEGF repebody, which is a small-sized protein binder consisting of leucine-rich repeat (LRR) modules. The anti-VEGF repebody selected through a phage-display was shown to have a high affinity and specificity for human VEGF. We demonstrate that this repebody effectively inhibits *in vitro* angiogenic cellular processes, such as proliferation and migration, by blocking the VEGF-mediated signaling pathway. The repebody was also shown to have a strong suppression effect on choroidal neovascularization (CNV) and vascular leakage *in vivo*. Our results indicate that the anti-VEGF repebody has a therapeutic potential for treating neovascular AMD as well as other VEGF-involved diseases including diabetic retinopathy and metastatic cancers.

## Introduction

Age-related macular degeneration (AMD) is the leading cause of severe vision loss and blindness among elderly people [1,2]. AMD is classified into two major types: dry (non-neovascular) and wet (neovascular) forms [3]. Wet AMD is known to be the most serious form and is caused by abnormal choroidal neovascularization (CNV). An excessive amount of vascular endothelial growth factor (VEGF) triggers the growth and leakage of abnormal blood vessels under the macular, resulting in irreversible loss of central vision [1,4–6]. In this context, many efforts have been made toward the development of anti-angiogenic therapies targeting VEGF for the treatment of wet AMD. Several types of anti-VEGF agents are clinically being used for the treatment of wet AMD, including bevacizumab, ranibizumab, aflibercept, and pegaptanib [7–9]. These drugs have been shown to slow the progression of AMD, and in some cases, improve vision acuity by suppressing angiogenesis. Of them, monoclonal antibodies, ranibizumab and bevacizumab, are widely used for wet AMD, displaying equivalent therapeutic efficacy in patients with neovascular AMD [10–12]. However, despite their widespread use, antibodies have some drawbacks such as a high production cost owing to the mammalian expression system, a tendency to aggregation, and a low tissue-penetration efficiency due to their large size [13,14]. In this regard, small-sized protein scaffolds have gained considerable attention as alternatives to monoclonal antibodies [15–17].

We previously developed a repebody scaffold with a small-size, which is composed of leucine-rich repeat (LRR) modules [18]. The repebody scaffold was shown to have desirable biophysical properties stemming from its modular architecture in terms of bacterial production, stability, and ease of design and engineering [19,20]. Herein, we present the development of an anti-human VEGF repebody as a potent anti-angiogenic agent. A repebody library was constructed by randomizing the variable sites on two modules, and anti-human VEGF repebodies were selected through a phage-display. Among them, repebody r-C2, with the highest binding affinity for human VEGF, was shown to effectively inhibit the angiogenic cellular processes by blocking the binding of VEGF to its receptor. We demonstrated a remarkable suppression effect of the repebody *in vivo* on the CNV formation and vascular leakage. The details are reported herein.

## Materials and Methods

### Construction of a phage-displayed library

Phagemid pBEL118N was used for insertion of a repebody library, as described in our previous study [18]. The repebody library was constructed by introducing random mutations into both modules 1 and 2 using PCR through the following mutagenic primers.

Module 1 (reverse): CGG CAG ATA CTG AAT GCC TTG CAC TGA TTT GAT ATC GGA *MNN MNN* CGC *MNN* GAT CTG GTC AAT

Module 2 (forward): GTG CAA GGC ATT CAG TAT CTG CCG AAT GTT CGT TAC CTG *NNK* ctg *NNK NNK* AAC AAA CTG CAT

The constructed library was cut using EcoRI and XhoI, and cloned into a pBEL118N vector followed by transformation into XL1-blue. The cells were grown in a 2XYT medium until the OD_600_ reached 0.6–0.7. To produce the phage-displayed library, the cells were infected with VCSM13 helper phage and grown overnight at 30°C. The phages were precipitated with 20% PEG solutions containing 200 mM of NaCl. The isolated phages were subjected to a standard panning process for the selection of anti-human VEGF repebodies.

### Selection of anti-human VEGF repebodies

To select anti-human VEGF repebodies, five rounds of bio-panning were carried out according to the standard protocols with minor modifications [21]. As a first step, 100 μg/mL of human VEGF was coated onto an immune-tube and left overnight at 4°C, followed by blocking with PBST containing 1% BSA for 2 hrs at 4°C. Phages of 10^12^ cfu/mL displaying the repebody library were incubated for 2 hrs at room temperature. Following five washings with TPBS for 5 min each, the immuno-tube was finally washed with PBS. The phages were eluted through incubation with 1 mL of 0.2 M glycine (pH 2.2), followed by neutralization using 60 μL of Tris-HCl (pH 9.0). The eluted phages were used to infect XL1-Blue cells, and the cells were plated onto 2XYT plates. After incubation overnight, the colonies were scraped from the plates and cultured. The phages were produced in a liquid culture through infection with the VCSM13 helper phage. The phages were purified and precipitated using a 20% PEG solution (200 mM NaCl). The purified phages were applied to the subsequent rounds of selection. Following five rounds of selection, individual colonies were seeded into a 96-deep well plate (Nunc), and cultured in 2XYT media for 6 hrs. The grown cells were infected with VCMS13 helper phages to produce repebody-displaying phages, followed by further incubation overnight at 30°C. After centrifugation at 3,500 rpm for 15 mins, the phages in supernatant were applied to a phage-based enzyme-linked immunosorbent assay (Phage-ELISA).

### Phage-based enzyme-linked immunosorbent assay (Phage-ELISA)

A 10 μg/mL of target proteins (human VEGF, PDGF, PlGF, and mouse VEGF) were immobilized on a 96-well maxisorp plate (Nunc) at 4°C overnight, followed by blocking with PBST containing 1% BSA, and a phage solution was added and incubated for 1 hr. Following three washings with PBST, a HRP-conjugated anti-M13 monoclonal antibody (1:5,000 dilution; GE healthcare) was incubated for 1 hr. After five washings with PBST, a solution of 3,3’,5,5’-tetramethylbenzidine (TMB) was added to each well for color development. The reaction was stopped by adding the same volume of 1 M sulfuric acid, followed by a measurement of the absorbance using an Infinite M200 plate reader (Tecan) at 450 nm.

### Competitive ELISA

Biotinylated-VEGF (20 nM) was incubated with 20 nM of Flt-1 and KDR (Biolegend) immobilized on a 96-well maxisorp plate in the presence of varying concentrations of r-C2 as a competitor. Streptavidin-HRP conjugates (Biolegend) were added and incubated for 1 hr, followed by the addition of a TMB solution for color development, and the absorbance was measured at 450 nm.

### Protein expression and purification

Repebodies were cloned into pET21a (Invitrogen) with a C-terminal His_6_ tag for purification, and transformed into an Origami B (DE3) cell. Cells were grown in an LB medium at 37°C until OD_600_ reached 0.5–0.6, followed by the addition of 0.5 mM IPTG and further incubation at 18°C for 18 hrs. The cells were harvested and disrupted using a sonicator, followed by centrifugation at 16,000 rpm at 4°C for 1 hr. Supernatant was filtered using a 0.4-μm syringe filter, and incubated with a Ni-NTA resin (Qiagen) for 1 hr at 4°C. Repebodies were eluted using an elution buffer containing 250 mM imidazole, and subjected to size exclusion chromatography (Superdex 200, GE healthcare) in a PBS (pH 7.4).

### Surface plasmon resonance (SPR) analysis

The binding affinity of the repebodies for VEGF was measured using a Biacore 3000 system (GE healthcare). VEGF (Gibco) was immobilized onto a CM5 chip (GE healthcare) using the EDC/NHS coupling method in a sodium acetate buffer (pH 5.5) flow, and the chip surface was blocked with 1 M of ethanolamine (pH 8.5). The reference channel was activated and blocked with ethanolamine. To evaluate the association and dissociation kinetics, varying concentrations of anti-VEGF repebodies and bevacizumab were injected into the PBS (pH 7.4) at a flow rate of 30 μL/min. The VEGF-immobilized chip surface was regenerated with 20 mM of NaOH between injections. All data were analyzed using BIAevaluation software (GE healthcare).

### Cell proliferation and viability assays

Human umbilical vascular endothelial cells (HUVECs) ordered from Lonza (Switzerland) were cultured in an EGM2 medium (Lonza) under 5% CO_2_ at 37°C, and the cells in passage 2–7 were used for cell proliferation and viability assays. For cell proliferation assay, 5.0 x 10^3^ HUVECs were cultured in an EBM-2 medium containing 1% fetal bovine serum (FBS; Gibco) in the presence of 20 ng/mL of VEGF and 2.5 μM of either bevacizumab (Roche) or a repebody. After 48 hrs, cell proliferation was measured using 3-(4,5-dimethylthiazol-2-yl)-2,5-diphenyltetrazolium bromide (MTT; Sigma-Aldrich), and an MTT assay was conducted according to the manufacturer’s protocols. As for cell viability assay, 5.0 x 103 HUVECs were seeded in a 96-well plate (SPL) and cultured in an EGM-2 medium containing varying repebody concentrations for 72 hrs. The cell viability was measured using an MTT assay.

### Migration assay

1.0 x 10^5^ HUVECs were added to a 24-well plate (SPL) in an EBM-2 medium containing 1% FBS, and incubated overnight. A monolayer of the cells was scratched using a loop and needle (SPL), followed by incubation in an EBM-2 medium containing 1% FBS in the presence of 20 ng/mL of VEGF and 2.5 μM of either a bevacizumab or a repebody for 4 hrs. Cells were fixed with 4% paraformaldehyde and stained with a crystal violet solution. The migration was quantified by counting the number of migrating cells in three randomly selected fields per well. The assay was performed three times independently.

### Western blot analysis

Serum-starved HUVECs were incubated with 50 ng/mL of VEGF and 2.5 μM of either bevacizumab or a repebody for 10 min. Cells were collected and lysed with PBS containing 1% Triton X-100. Equal amounts of proteins were loaded into the wells of the SDS-PAGE gel and transferred to nitrocellulose membrane filters for 2 hrs at 100 V. The membranes were blocked with PBST containing 1% BSA for 1 hr at room temperature, and probed with primary anti-ERK and anti-phospho ERK antibodies (Santa Cruz Biotechnology and Cell Signaling Technology) for 1 hr at room temperature. The membranes were washed three times with PBST, followed by incubation with a secondary antibody for 1 hr at room temperature. Following three washings with PBST, immuno-reactive bands were detected using an ECL system (Millipore), and photographed using an Odyssey (Li-COR) imaging system. The data were quantified using Image J software.

### CNV mouse model

All animals and experiment procedures were maintained in accordance with the ARVO Statement for the Use of Animals in Ophthalmic and Vision Research and approved by the Institutional Animal Care and Use Committee of Soonchunhyang University Bucheon Hospital. Eight-week old C57BK/6J mice (Orient, Korea) were housed in a temperature- and humidity-controlled room under a 12-hrs light/dark cycle. Laser photocoagulation (200-μm spot size, 0.04-sec duration, 150-mW laser power) was performed with the slit lamp delivery system of a PASCAL diode laser (Topcon Medical Laser Systems, Inc., Santa Clara, CA). Three laser spots were distributed in a concentric pattern around the optic nerve head of the mouse eye at 2, 6, and 10 o’clock. After 7 days, PBS (control) and varying concentrations of a repebody were injected intravitreally. The mice were raised for an additional 7 days. Animals were anesthetized by intraperitoneal (IP) injection of a mixture of 40 mg/kg of zolazepam/tiletamine (Zoletil; Virbac, Carros Cedex, France) and 5 mg/kg of xylazine (Rompun; Bayer Healthcare, Leverkusen, Germany), followed by pupil dilation with 0.5% tropicamide and 2.5% phenylephrine (Mydrin-P; Santen, Emeryville, CA). Fluorescein angiography (FAG) and indocyanine green angiography (ICGA) images were taken using a confocal scanning laser ophthalmoscope (Heidelberg Retina Angiograph 2, Germany).

### Immunohistochemistry analysis

Mice were deeply anesthetized by IP injection of 4:1 mixture of Zolazepam/Tiletamine (80 mg/kg), and Xylazine (10 mg/kg), and were intracardially perfused with 0.1 M phosphate buffer (PB) containing 1,000 U/mL of heparin, followed by 4% paraformaldehyde (PFA) in 0.1 M PB. Eyeballs were enucleated. The harvested choroid/sclera complex was fixed and prepared using four equidistant cuts. Samples were blocked with 5% goat serum in PBST, and incubated with the primary anti-CD31 antibody (1:100, BD PharMingen) overnight at 4°C. On the following day, the samples were incubated with a secondary antibody for 1 hr at room temperature in the dark, followed by an analysis using a confocal microscope (LSM 510 META; Carl Zeiss, Germany) at a magnification of 400X. Images were captured using image-capture software (LSM Image Browser; Carl Zeiss, Germany).

### Statistical analysis

The values herein are presented as the mean ± SD, and significant differences between the means were determined using an unpaired Student’s *t* test. The statistical significance was set at P < 0.05.

## Results and Discussion

### Selection of anti-human VEGF repebodies

**Fig 1:**
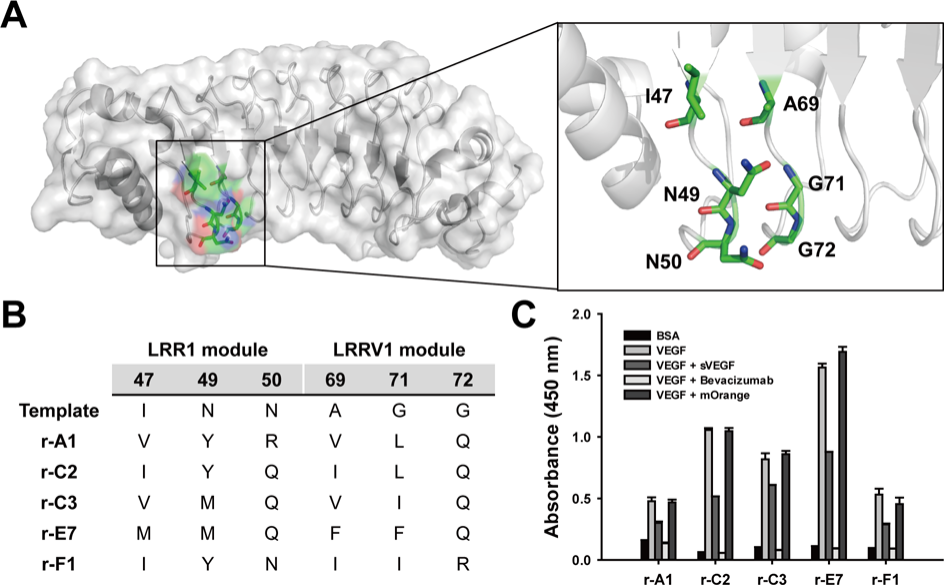
Selection of anti-human VEGF repebodies using a phage display. (**A**) Sites for introducing mutations on a repebody for phage-displayed library construction. (**B)** Amino acid sequences of the five isolated repebodies. (**C)** Identification of binding epitopes for selected repebodies using a competitive phage-ELISA. Phage-displayed repebodies were incubated with human VEGF (1 μg/mL) coated on a 96-well plate, followed by addition of 100 μg/mL VEGF, bevacizumab and mOrange. mOrange was used as a negative competitor.

We constructed a repebody library by randomizing six variable sites on LRR1 and LRRV1 modules (**Fig 1A**), and selected ten repebody clones showing a significant signal increase (signal to noise > 10) for human VEGF through a phage display and standard panning process. We conducted multiple sequence alignments to obtain insight into the binding interface based on amino acid sequences of the selected repebodies (**S1 Fig**). Most of the hydrophobic amino acids were conserved at more than half of the sites, and some aromatic amino acids were also conserved at positions 49, 69, and 71. Interestingly, polar or charged residues were observed in the third position of both modules. Of them, for further test, we chose five clones with distinct sequences showing high signals in the phage-ELISA (**Fig 1B**). To predict the binding epitopes of the selected repebodies, we conducted a competitive phage-ELISA using bevacizumab as a competitor. Bevacizumab was revealed to have the epitope at the binding interface with human VEGF [22]. All repebodies were shown to exhibit significantly decreased signals only in presence of bevacizumab (**Fig 1C**), implying that the five repebodies shared an epitope with bevacizumab. Based on this result, it is expected that the selected repebodies may effectively inhibit the binding of VEGF to its receptors.

### Biochemical properties of selected repebodies

To investigate the biochemical properties of the selected repebodies, we expressed the repebodies in *E*.*coli* followed by purification using a Ni-NTA agarose resin and subsequent size-exclusion chromatography (SEC). The size-exclusion chromatogram of the repebodies resulted in a monomeric peak as in SDS-PAGE analysis (**S2 Fig**). Circular dichroism (CD) spectrometry showed a similar far-UV spectrum to the template scaffold, indicating that the selected repebodies have almost the same secondary structures as the template scaffold (**S3A Fig**). We next determined the binding affinities of the selected repebodies against human VEGF using surface plasmon resonance (SPR). The repebodies were shown to have binding affinities ranging from 10 to 357 nM (**Table 1**). Considering the binding epitope and affinity of the repebodies, we chose r-C2 to investigate *in vitro* and *in vivo* inhibitory effects.

**Table 1:** Kinetic rate constants and binding affinities of repebodies for human VEGF.

Repebody	*k*_*a*_ [M^-1^s^-1^]	*k*_*d*_ [s^-1^]	*K*_*D*_ [M]
**r-A1**	7.88 x 10^5^	0.205	2.60 x 10^−7^
**r-C2**	9.02 x 10^5^	0.009	1.06 x 10^−8^
**r-C3**	7.90 x 10^5^	0.282	3.57 x 10^−7^
**r-E7**	1.04 x 10^6^	0.281	2.69 x 10^−7^
**r-F1**	1.31 x 10^6^	0.106	8.04 x 10^−8^

**Fig 2:**
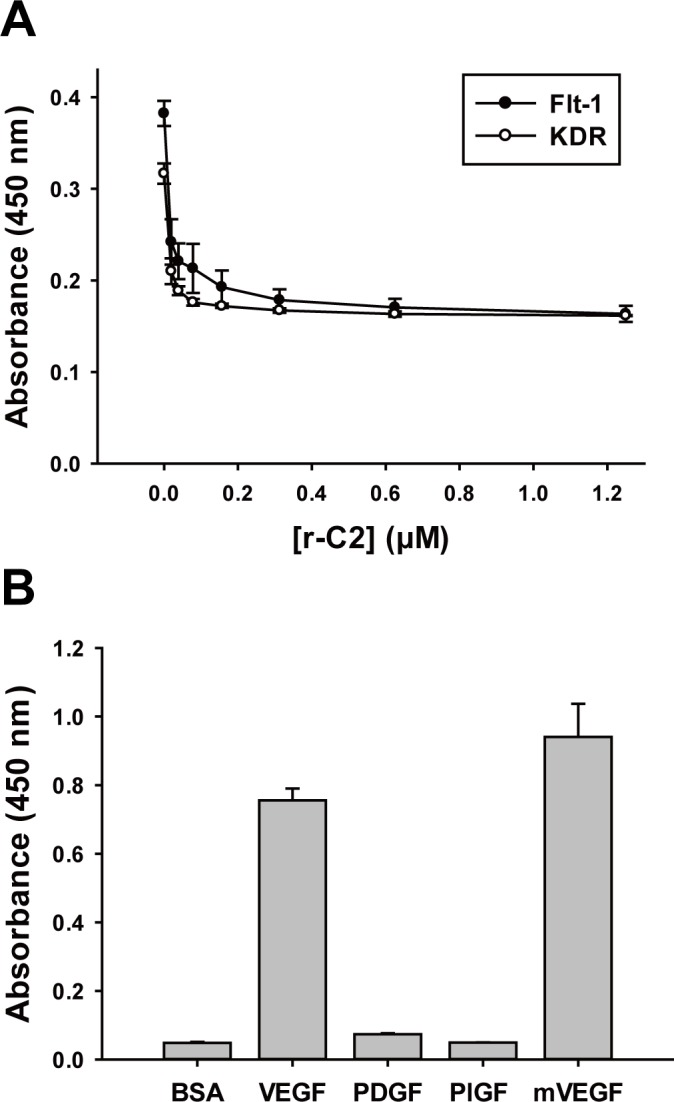
Inhibitory effect of selected repebody on binding of VEGF to its receptors. (**A**) Inhibitory effect of r-C2 on the binding of VEGF to its receptors (Flt-1 and KDR). (**B**) Analysis of cross-reactivity of r-C2 against VEGF-family proteins (PDGF and PlGF) using ELISA. The species-specificity was tested in the same manner.

It is known that human VEGF stimulates angiogenesis and vascular permeability by mainly interacting with two VEGF receptors, Flt-1 and KDR [23,24]. VEGF binds to Flt-1 much more strongly than to KDR, but its effect on the activation of the signaling pathways is weak [25]. For effective inhibition of VEGF-mediated signaling pathways, a VEGF-specific repebody is required to prevent VEGF from binding to both receptors. To test this, we conducted a competitive ELISA using r-C2 as a competitor against biotinylated-VEGF toward Flt-1 and KDR. As shown in **Fig 2A**, r-C2 was observed to have an inhibitory effect on the interaction of VEGF with its receptors in a concentration-dependent manner. We next examined the specificity of r-C2 against VEGF family members, such as platelet-derived growth factor (PDGF) and placental growth factor (PlGF). Human VEGF has 23% and 43% amino acid sequence similarities with PDGF and PlGF, respectively. As a result of a phage- ELISA, r-C2 was shown to have a high specificity for human VEGF, exhibiting a negligible interaction with other family members (**Fig 2B**). In a species-specificity test, r-C2 displayed cross-reactivity against mouse VEGF, which shares an 87% amino acid sequence similarity with human VEGF.

### Inhibitory effect by a repebody on VEGF-mediated signaling pathway

**Fig 3:**
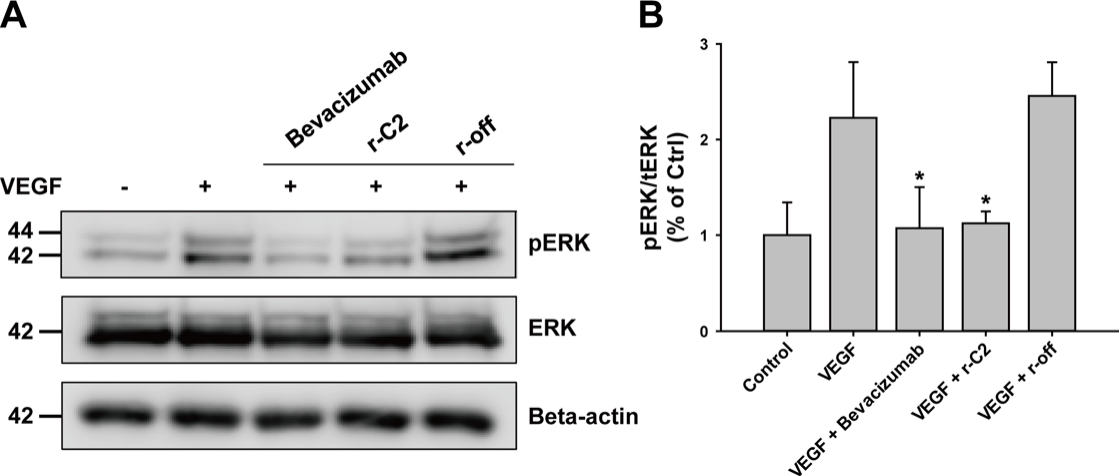
Inhibitory effect of a repebody on VEGF-mediated ERK activation. (**A**) Western blot analysis of inhibitory effect of r-C2 on ERK phosphorylation. Serum-starved HUVECs were incubated with VEGF (20 ng/mL) in the absence or presence of 2.5 μM of either a repebody or bevacizumab for 10 min. The total cell lysates were subjected to immunoblot analysis for phosphorylated and total ERK using the respective antibodies. The band intensities were measured using Image J software. An antibody against beta-actin was used as a control. (**B**) Quantitative analysis of the inhibitory effect by r-C2 on ERK activation based on (A). The degree of inhibition was presented as the ratio of the phosphorylated ERK level to the total ERK. *P < 0.05 compared with incubation using VEGF only.

The binding of VEGF to its receptors induces phosphorylation, leading to the activation of many signal-transduction pathways, and consequently, angiogenesis and vasculogenesis [26]. One of the major pathways is an extracellular signal-regulated kinase (ERK)-related signal cascade [27]. To examine the inhibitory effect of r-C2 on VEGF-mediated signaling, we analyzed the level of phosphorylated ERK in serum-starved human vascular endothelial cells (HUVECs) using a western blot analysis. The cells were incubated with human VEGF (50 ng/mL) in the presence of 2.5 μM of either a repebody or bevacizumab for 10 min, and the phosphorylated ERK level was then measured. As shown in **Fig 3**, the addition of r-C2 significantly decreased the phosphorylated ERK levels in the cells, showing an effect comparable to that of bevacizumab. On the other hand, the off-target repebody had a negligible effect on the level of ERK phosphorylation. It is likely that r-C2 effectively inhibits the VEGF-mediated signaling pathways by blocking the binding of VEGF to its receptors.

### *In vitro* suppression of angiogenic process by a repebody

**Fig 4:**
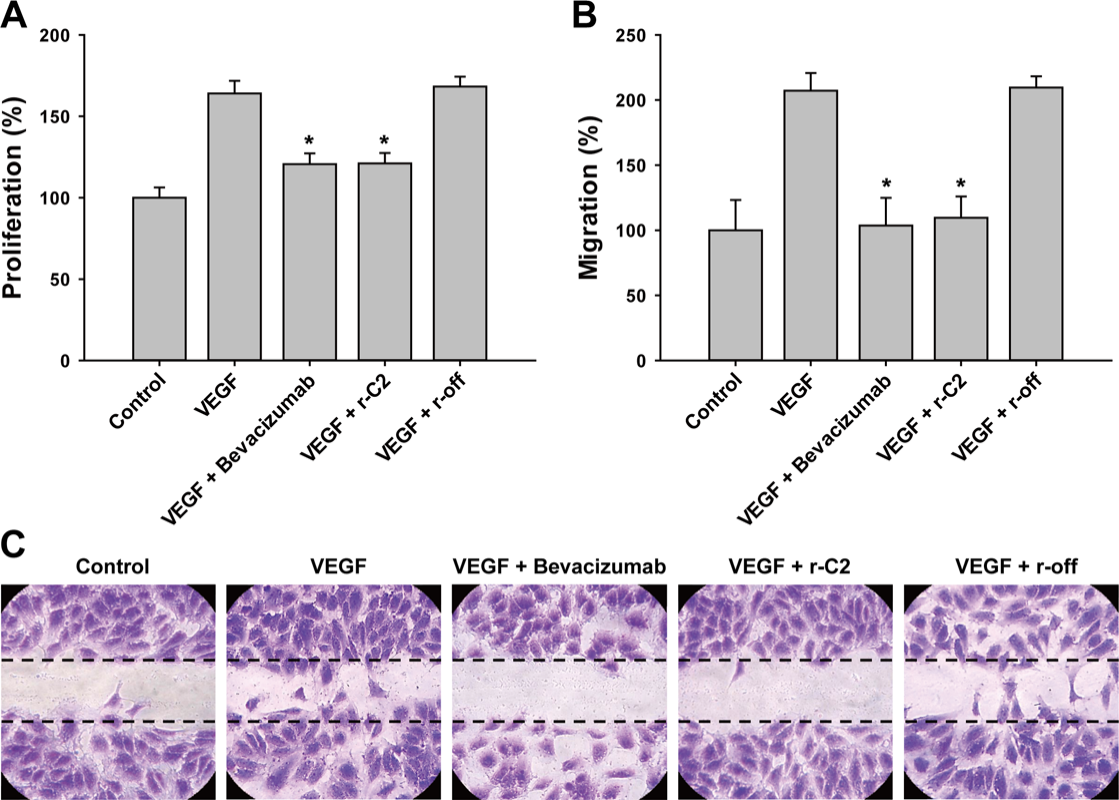
Suppression of angiogenic cellular process by a repebody. (**A**) Effect of r-C2 on VEGF-induced proliferation of HUVECs. Cells were incubated with VEGF (20 ng/mL) in the presence of 2.5 μM of either a repebody or bevacizumab for 48 hrs. *P < 0.05 compared with incubation using VEGF only. (**B**) Inhibition of VEGF-induced cellular migration by r-C2. HUVECs on the plate were scratched using a needle and incubated with VEGF in the presence of either a repebody or bevacizumab. The dotted line indicates a scratched area. The number of cells that migrated across the line were counted and analyzed. This experiment was performed in triplicate. *P < 0.05 compared with incubation using VEGF only. (**C**) Images of endothelial cellular migration assay. HUVECs were stained with a 0.05% crystal violet solution.

**Fig 5:**
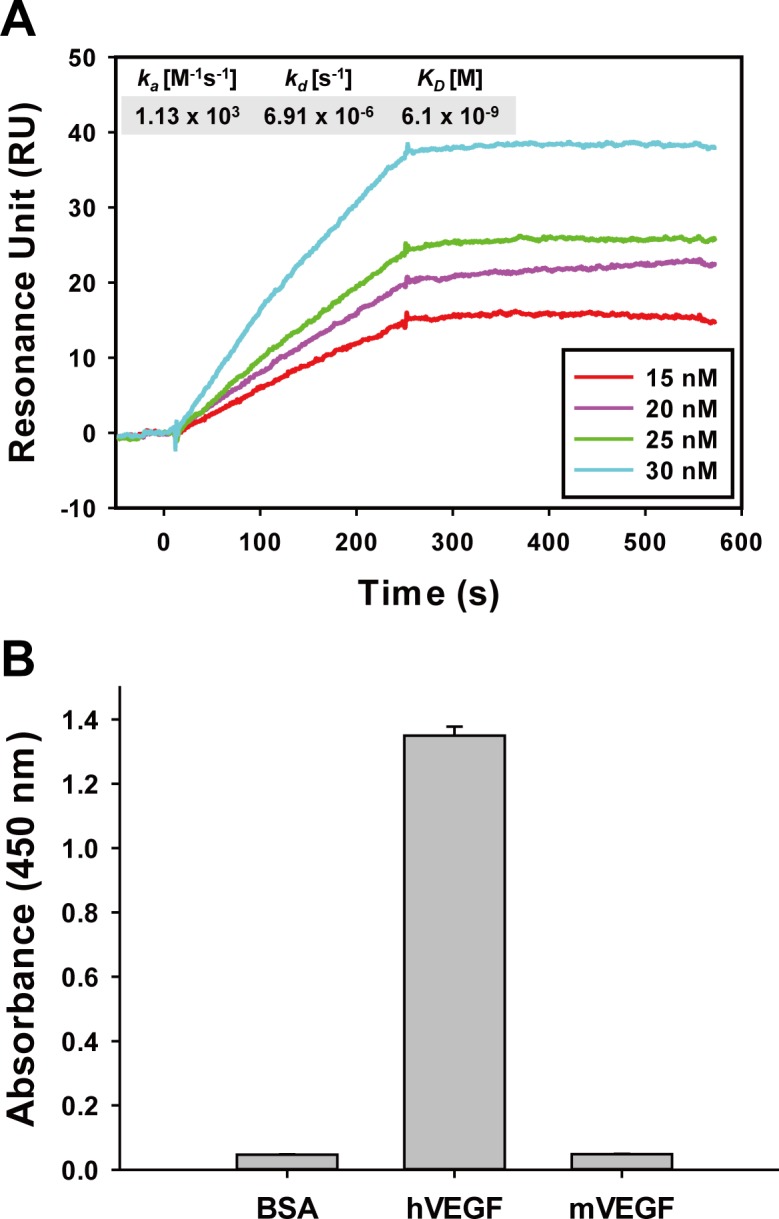
SPR analysis and species-specificity of bevacizumab. (**A**) SPR sensorgram and kinetic constants of bevacizumab for human VEGF. (**B**) Species-specificity of bevacizumab against mouse VEGF by ELISA.

VEGF is known to induce vascular permeability and angiogenesis by promoting cellular proliferation and migration [28]. We checked to determine whether r-C2 suppresses the cellular proliferation and migration in HUVECs. Cells were incubated with human VEGF (20 ng/mL) in the presence of 2.5 μM of either r-C2 or bevacizumab. As shown in **Fig 4A**, r-C2 effectively repressed the endothelial cell proliferation, exhibiting a comparable effect as bevacizumab. In addition, r-C2 significantly suppressed the cellular migration (**Fig 4B and 4C**). It was also shown that the binding epitope and affinity of r-C2 for VEGF is similar to those of bevacizumab (**Fig 5A**) [29]. Therapeutic agents should have a low level of toxicity and high stability [30,31]. We checked the endothelial cellular toxicity of r-C2 using HUVEC cells. The cells were incubated with varying concentrations of the repebody from 1 nM to 100 μM for 72 hrs, and the viability of the cells was analyzed. As a result, a negligible cellular toxicity was observed even at 100 μM of r-C2 (**S5 Fig**). Stability of r-C2 was examined by measuring the melting temperature using CD spectrometry (**S3B Fig**). The melting temperature of r-C2 was estimated to be 82°C, implying a high stability of the repebody.

### *In vivo* anti-angiogenic effect by a repebody

**Fig 6:**
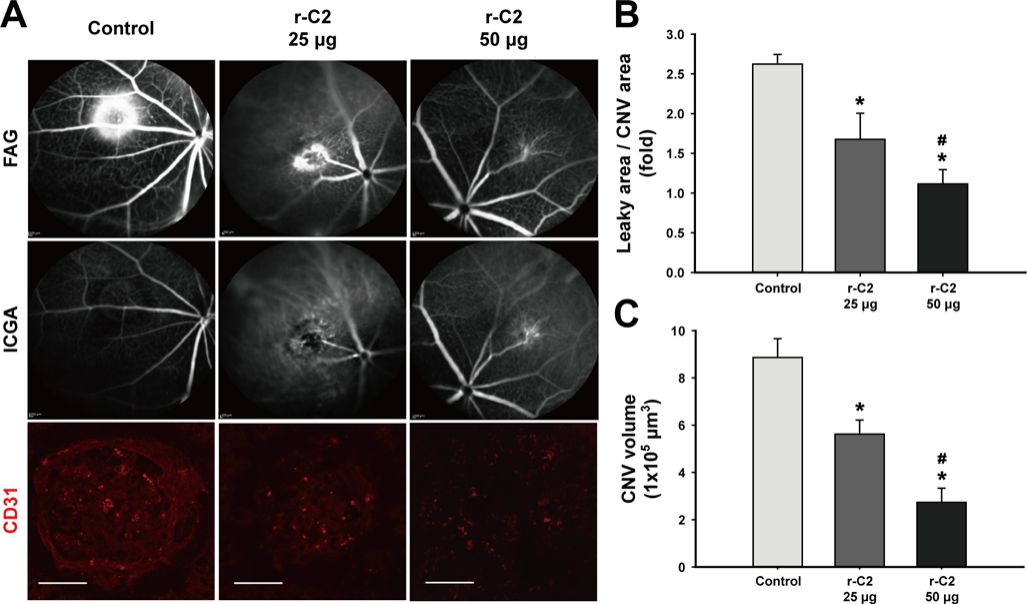
Anti-angiogenic potency of a repebody in the CNV mouse model. (**A**) Fluorescein angiography (FAG) and indocyanine green angiography (ICGA) images of laser photocoagulation sites after treatment with varying concentrations of r-C2. Laser-induced CNV mice were treated with 25 μg and 50 μg of r-C2 intravitreally seven days after laser photocoagulation. Phosphate buffered saline was used as a control. Seven days later, FAG and ICGA were conducted and choroid/sclera complexes were harvested. Late-phage (6 minutes) FA was carried out to detect vascular leakages, and ICGA and CD31 staining were conducted to measure the extent of CNV. Scale bars: 50 μm. (**B**) The ratios of leaky area to CNV area when treated with 25 μg and 50 μg of r-C2. The leaky area was estimated by measuring the total hyperfluorescent area using FA, and the CNV area was calculated using ICGA. (**C**) Change in CNV volume by treatment with 25 μg and 50 μg of r-C2. The CNV volume was calculated based on the total measured CD31^+^ CNV volume. For each group, n = 5. *P < 0.05 vs. control. ^#^P < 0.05 vs. 25 μg of r-C2.

We further assessed the potential therapeutic effect of r-C2 in CNV mice as a wet AMD model. CNV was induced through laser-driven photocoagulation in eight-week old mice, and its formation was confirmed through the generation of a bubble that occurred owing to the rupture of the Bruch’s membrane, which is known to be a typical feature in a CNV model. After seven days, two different doses of r-C2 (25 μg and 50 μg) and PBS were injected intravitreally. PBS was used as a control. At seven days after injection, fluorescein angiographic (FAG) and indocyanine green angiography (ICGA) images were analyzed. At the same time, retinal pigment epithelium-choroid complexes were examined through an immuno-histochemical analysis using an anti-CD31 antibody. As shown in **Fig 6A**, vascular leakage caused by the new blood vessel formation was clearly observed at the lase-induced photocoagulation sites. The intravitreal injection of 25 μg of r-C2 was shown to decrease the vascular leakage by 36%, and the vascular leakage further decreased up to 58% with an increased dose of 50 μg (**Fig 6B**). Similarly, the intravitreal administration of 50 μg of r-C2 significantly reduced the CNV volume up to 69% in a dose-dependent manner (**Fig 6C**). Based on this result, it is likely that the anti-human VEGF repebody had a strong anti-angiogenic effect in the CNV mouse model used. Comparison of the *in vivo* efficacy with bevacizumab was impossible because bevacizumab has no cross-reactivity toward mouse VEGF (**Fig 5B**) [32].

## Conclusions

We have shown that an anti-human VEGF repebody effectively suppresses the CNV formation and vascular leakage *in vivo* by blocking the binding of VEGF to its receptors. AMD is the leading cause of severe vision loss and blindness among elderly people. The VEGF-mediated signaling process is known to play a crucial role in angiogenesis, causing retinopathy. The elevated levels of VEGF cause neovascularization and vascular leakage in a variety of ocular diseases. As therapeutic agents for AMD, anti-VEGF monoclonal antibodies and their fragment forms have been developed and clinically used. In addition, a Fc-fused VEGF receptor and aptamer were approved for clinical use. Our previously developed repebody scaffold is composed of LRR modules, showing a desirable property as an alternative to immunoglobulin antibodies. Modular architecture of the repebody enabled easy construction of a module-based library and selection of an anti-human VEGF repebody through a phage display. The selected repebody r-C2 exhibited a high specificity toward human VEGF, effectively inhibiting the angiogenic processes both *in vitro* and *in vivo*. Our results demonstrated the potential of r-C2 as a therapeutic agent for wet AMD.

As a protein scaffold, the repebody was shown to have unique properties arising from its modular architecture, including ease of engineering and high stability against pH, heat, and proteolytic digestion [18]. Furthermore, the repebody is easy to produce using a bacterial expression system. Owing to its small size, the repebody was revealed to have a high tissue penetration compared to a monoclonal antibody [33]. Considering the effective inhibitory effect of r-C2 on CNV formation and vascular leakage *in vivo*, an anti-human VEGF repebody can be further developed as a therapeutic agent for various VEGF-mediated ocular neovascular diseases such as wet AMD, diabetic retinopathy, and other retinal disorders.

## Supporting Information

S1 Fig Web logo for conserved amino acid residues at mutation sties of the selected clones. (TIF)

S2 Fig Size-exclusion chromatograms and SDS-PAGE analysis for selected anti-human VEGF repebodies. (TIF)

S3 Fig CD analysis of selected repebodies. (**A**) CD spectra of selected repebodies were measured from 190 nm to 270 nm at 25°C using a J-815 CD spectrometer. (**B**) Molar ellipticity at 222 nm was measured with a gradual increase (1°C/min) of temperature from 25°C to 90°C. The melting temperature of r-C2 was estimated to be 82°C using denaturation analysis program (Jasco).
(TIF)

S4 Fig SPR analysis of selected anti-human VEGF repebodies. Sensorgrams of selected repebodies. Concentrations of injected repebodies were indicated in inset.
(TIF)

S5 Fig Endothelial cellular toxicity test of r-C2. HUVECs were incubated with varying concentrations of r-C2 ranging from 1 nM to 100 μM for 72 hrs at 37°C, and their viabilities were measured by MTT assay.
(TIF)
