# Supplementary figures and images for: Anti-Human VEGF Repebody Effectively Suppresses Choroidal Neovascularization and Vascular Leakage
Source: PLoS One. 2016 Mar 25;11(3):e0152522. doi: 10.1371/journal.pone.0152522 (PMC4807815; doi:10.1371/journal.pone.0152522)

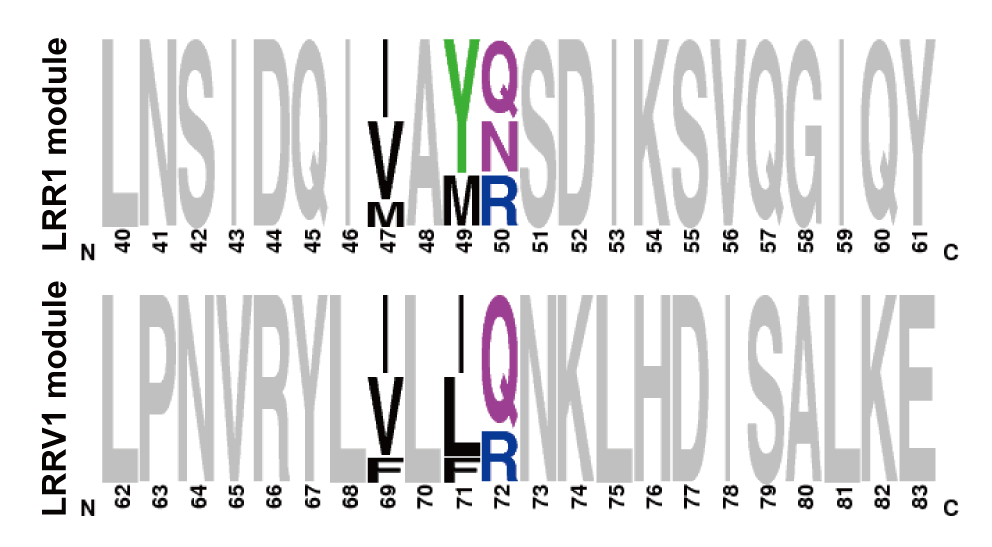

Supplement: S1 Fig — (TIF) [file pone.0152522.s001.tif]

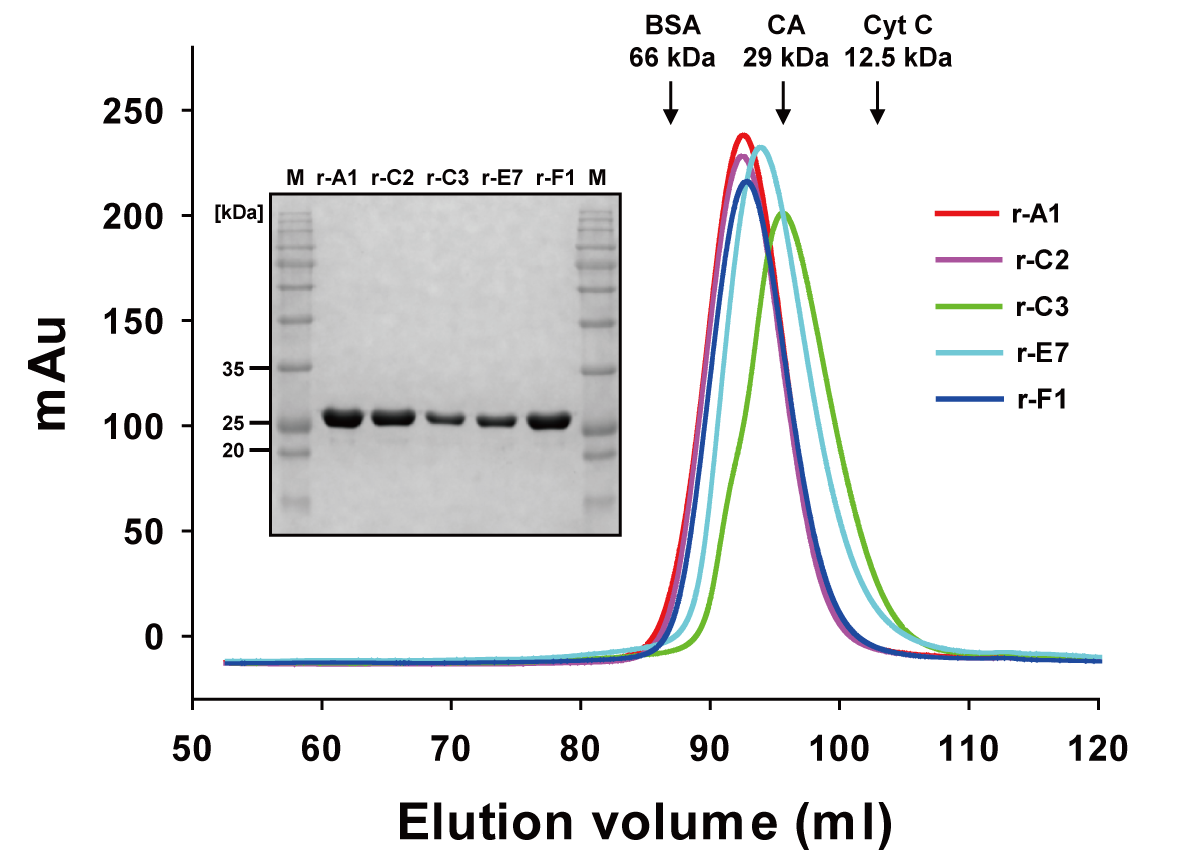

Supplement: S2 Fig — (TIF) [file pone.0152522.s002.tif]

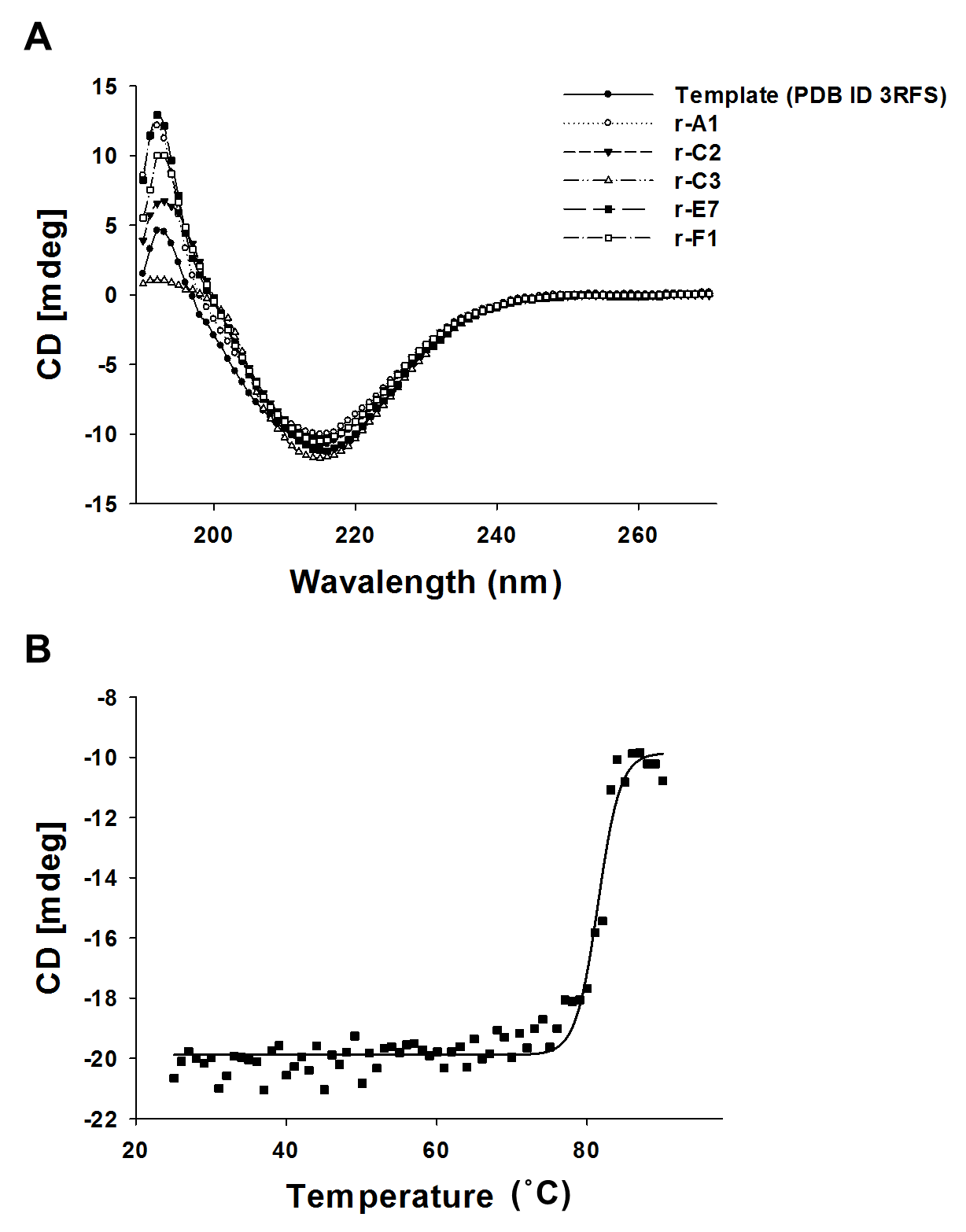

Supplement: S3 Fig — (A) CD spectra of selected repebodies were measured from 190 nm to 270 nm at 25°C using a J-815 CD spectrometer. (B) Molar ellipticity at 222 nm was measured with a gradual increase (1°C/min) of temperature from 25°C to 90°C. The melting temperature of r-C2 was estimated to be 82°C using denaturation analysis program (Jasco). (TIF) [file pone.0152522.s003.tif]

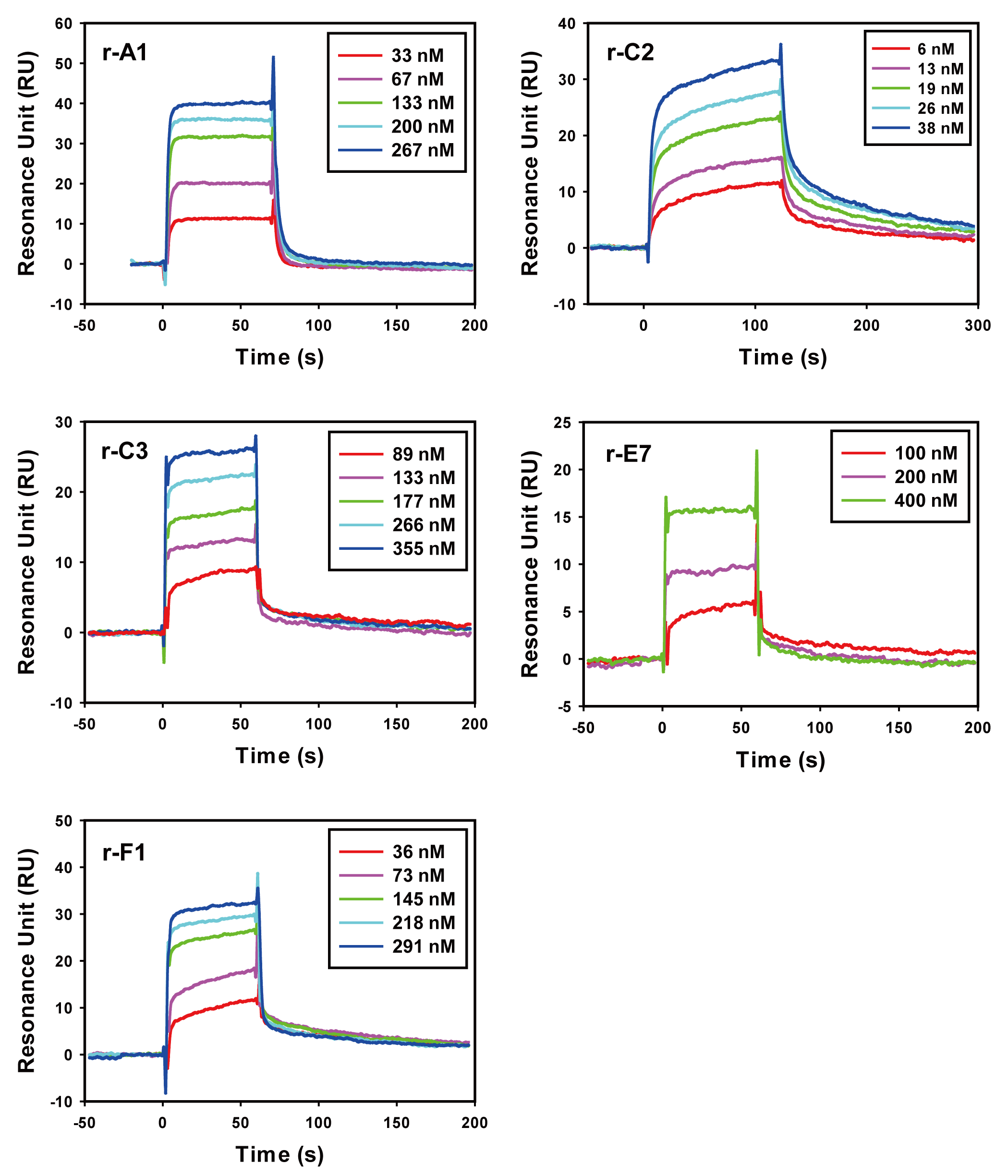

Supplement: S4 Fig — Sensorgrams of selected repebodies. Concentrations of injected repebodies were indicated in inset. (TIF) [file pone.0152522.s004.tif]

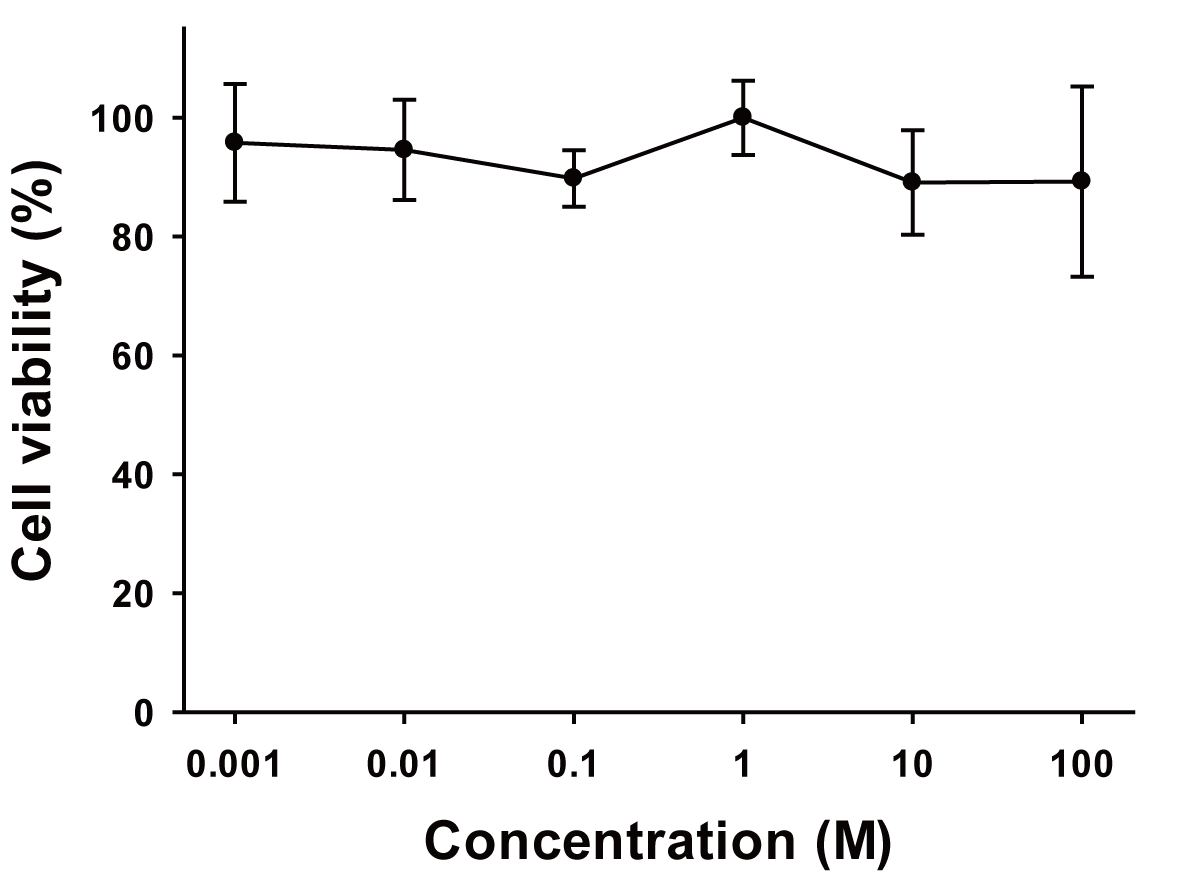

Supplement: S5 Fig — HUVECs were incubated with varying concentrations of r-C2 ranging from 1 nM to 100 μM for 72 hrs at 37°C, and their viabilities were measured by MTT assay. (TIF) [file pone.0152522.s005.tif]
